# An anatomical approach to the tarsal tunnel syndrome: what can ankle’s medial side anatomy reveal to us?

**DOI:** 10.1186/s13047-023-00682-4

**Published:** 2023-11-14

**Authors:** Jorge Gomes Lopes, André Rodrigues-Pinho, Maria Abreu Neves, Filipe Fonseca Pinto, Miguel Relvas-Silva, Luísa Vital, Francisco Serdoura, António Nogueira-Sousa, Maria Dulce Madeira, Pedro Alberto Pereira

**Affiliations:** 1 Orthopedics and Traumatology Unit; São João University Hospital Center, Porto, Alameda Prof. Hernâni Monteiro, 4200-319 Porto, Portugal; 2 Unit of Anatomy, Department of Biomedicine, Faculty of Medicine, University of Porto, Alameda Prof. Hernâni Monteiro, 4200-319 Porto, Portugal; 3 NeuroGen Research Group, Center for Health Technology and Services Research (CINTESIS), Rua Dr. Plácido da Costa, 4200–450 Porto, Portugal; 4 CINTESIS@RISE, Faculty of Medicine, University of Porto, Alameda Prof. Hernâni Monteiro, 4200-319 Porto, Portugal

**Keywords:** Tarsal tunnel syndrome, Heel pain syndrome, Medial ankle anatomy, Tibial nerve, Medial plantar nerve, Lateral plantar nerve, Baxter’s nerve

## Abstract

**Background:**

The heel is a complex anatomical region and is very often the source of pain complaints. The medial heel contains a number of structures, capable of compressing the main nerves of the region and knowing its anatomical topography is mandatory. The purpose of this work is to evaluate if tibial nerve (TN) and its main branches relate to the main anatomical landmarks of the ankle’s medial side and if so, do they have a regular path after emerging from TN.

**Methods:**

The distal part of the legs, ankles and feet of 12 Thiel embalmed cadavers were dissected. The pattern of the branches of the TN was registered and the measurements were performed according to the Dellon–McKinnon malleolar-calcaneal line (DML) and the Heimkes Triangle (HT).

**Results:**

The TN divided proximal to DML in 87.5%, on top of the DML in 12,5% and distal in none of the feet. The Baxter’s nerve (BN) originated proximally in 50%, on top of the DML in 12,5% and distally in 37.5% of the cases. There was a strong and significant correlation between the length of DML and the distance from the center of the medial malleolus (MM) to the lateral plantar nerve (LPN), medial plantar (MPN) nerve, BN and Medial Calcaneal Nerve (MCN) (ρ: 0.910, 0.866, 0.970 and 0.762 respectively, *p* <  0.001).

**Conclusions:**

In our sample the TN divides distal to DML in none of the cases. We also report a strong association between ankle size and the distribution of the MPN, LPN, BN and MCN. We hypothesize that location of these branches on the medial side of the ankle could be more predictable if we take into consideration the distance between the MM and the medial process of the calcaneal tuberosity.

## Background

The heel is a complex anatomical region and is very often the source of pain complaints [1–3]. Diagnosing the etiology of this pain is not always easy [2, 4]. A specific cause can only be found in 60 to 80% of the cases [2, 5].

In order to better encounter an answer, one must be aware of the importance of putting together a good knowledge of the anatomy involved, a detailed history, thorough physical examination and directed complementary exams.

**Fig. 1:**
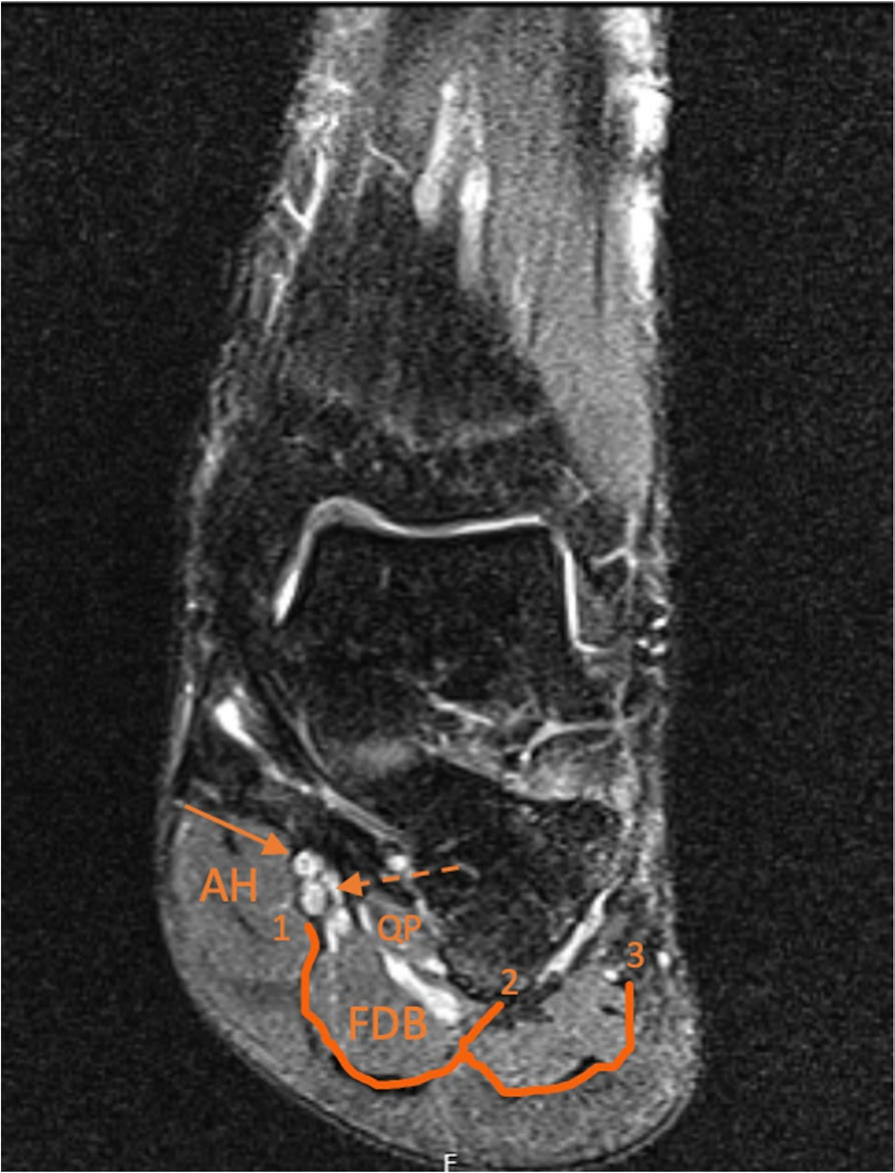
Coronal T2 weighted magnetic resonance image of a 39 year old male left ankle and foot. 1: Medial Septum; 2: Intermediate septum; 3: Lateral Septum. AH: Abdutor Hallucis; FDB: Flexor Digitorum Brevis; QP: Quadratus Plantae; Dashed Arrow: lateral plantar nerve (LPN). Non-dashed arrow: medial plantar nerve (MPN)

One important cause of chronic heel pain is neurologic compression and, among them, the tarsal tunnel syndrome (TTS) [6]. It represents an entrapment neuropathy of the tibial nerve (TN) and its branches on the medial side of the ankle [2, 5, 6]. The cause of the entrapment is still debated. In 1987, Heimkes et al. described a “proximal” and a “distal” tarsal tunnel (TT) according to the location [7]. The first, refers to the compression under flexor retinaculum (FR), the second, to the entrapment of one of the branches of the TN under the abductor hallucis (AH) and surrounding fascia [7]. The same authors described that both the medial plantar (MPN) and the lateral plantar (LPN) nerves had a fibrous tunnel each, taking them under the AH [7]. Several anatomical studies shed some light into those observations, outlining the role of these osteofibrous tubes and the intermuscular septae [5, 6, 8, 9]. Ling and Kumar described three vertical fascial septae on the plantar aspect of the foot, namely, medial, lateral and intermediate [9]. More recently, Singh and Kumar, validated the three septae in the sole of the foot, the medial and lateral observed in all feet (*n* = 19) and an intermediate septum that was well developed in 47.4% of the specimens (*n* = 9), indistinct in 26,3% (*n* = 5), and absent in 26,3% (n = 5) of the specimens [5]. The medial septae, a dorsal expansion of the medial border of the plantar fascia that separates the AH from the flexor digitorum brevis and quadratus plantae (flexor accessorius) (Fig.1), was considered the most important cause of entrapment neuropathy, beside the FR, in heel pain syndrome [5, 8]. This was also the site described for a potential compression of the first branch of the LPN, the Baxter’s Nerve (BN) [10].

Surgical treatment for TTS is reserved to whom does not respond to conservative measures [2]. However, the decision of how to operate is controversial. Good and excellent results were described with releasing the FR plus the distal TT (MPN, LPN, BN) [1] but some advocate the need to also dissect the medial calcaneal nerve (MCN) [11].

Being able to diagnose and treat the TTS demands a good understanding of the topographical relationship between neural structures and possible compression points in the medial heel region. The current literature is still not sufficient to provide that knowledge and we aimed to give a greater insight regarding the anatomical landmarks behind TTS.

## Materials and methods

The distal part of the leg, ankles and feet (12 left and 12 right) of 12 Thiel embalmed cadavers (7 males, 5 females) were used in this study. The cadavers derived from body donation with informed consent, written and signed by the donator himself (Portuguese Decree-law number 274/99). Cadavers were received and embalmed at the Unit of Anatomy, Department of Biomedicine, Faculty of Medicine, University of Porto. The subjects included were all Caucasian. The male donors had an average age of 78 years and an average height of 172 cm. The female donors were, on average, 63 years old and 164 cm tall.

Exclusion criteria for the use of a specimen were visible signs of previous ankle or foot trauma or surgery, pathological ankle or foot deformities or space-occupying lesions.

**Fig. 2:**
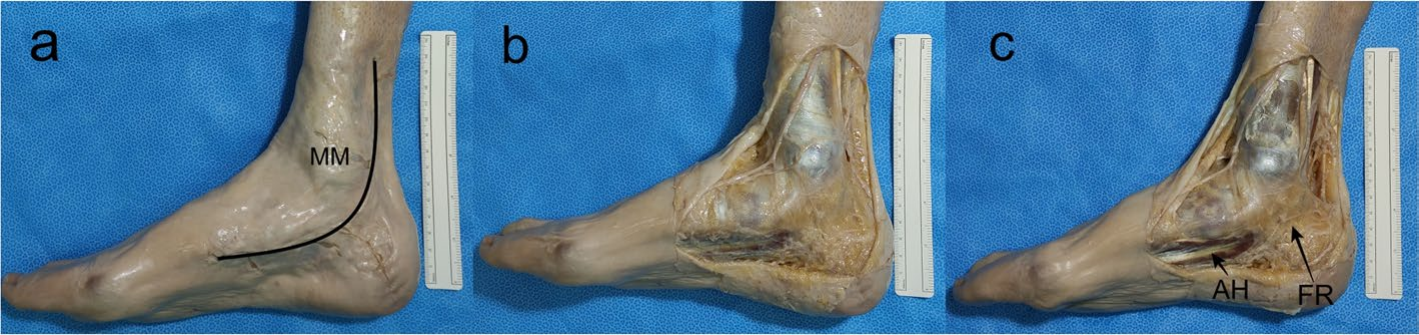
A posteromedial approach to the ankle was used. The skin incision started approximately 8 cm proximal to the medial malleolus (MM) and extended nearly to the level of the talocalcaneonavicular joint (**a**). Small incisions approximately perpendicular to it were done to facilitate the retraction of the skin. The skin was carefully retracted leaving the superficial fascia, intact. Then, the superficial fascia was carefully removed to preserve the vascular and nervous structures lying in it (**b**). The deep fascia, and a band-shaped thickening of it, the flexor retinaculum (FR), were identified. The deep fascia was removed leaving the FR intact and the proximal part of the abdutor hallucis (AH) was identified and dissected (**c**)

**Fig. 3:**
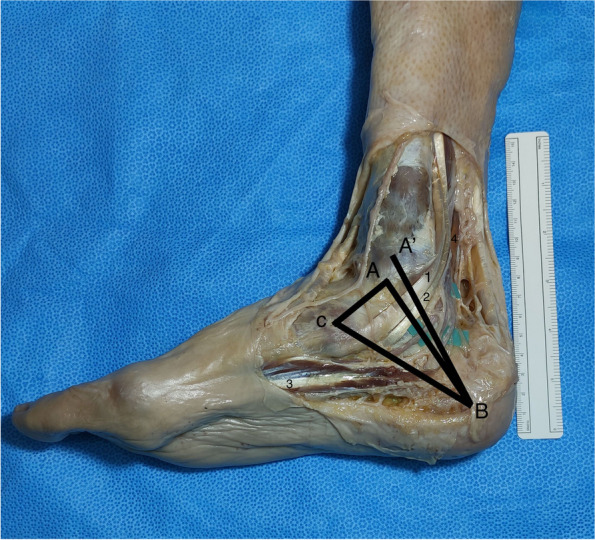
Medial view of the right distal part of the leg, ankle and foot. 1 – Tibial posterior tendon; 2 – Flexor digitorum longus tendon; 3 – Abdutor Hallucis; 4 – Flexor Hallucis Longus. Heimkes Triangle (HT) with the following vertices: A – the tip of medial malleolus (MM); B – the tip of the medial process of the calcaneal tuberosity at its greatest distance from the MM; C – the tuberosity of the navicular. The Dellon–McKinnon malleolar-calcaneal line (DML): A’ – the center of the MM to B

**Fig. 4:**
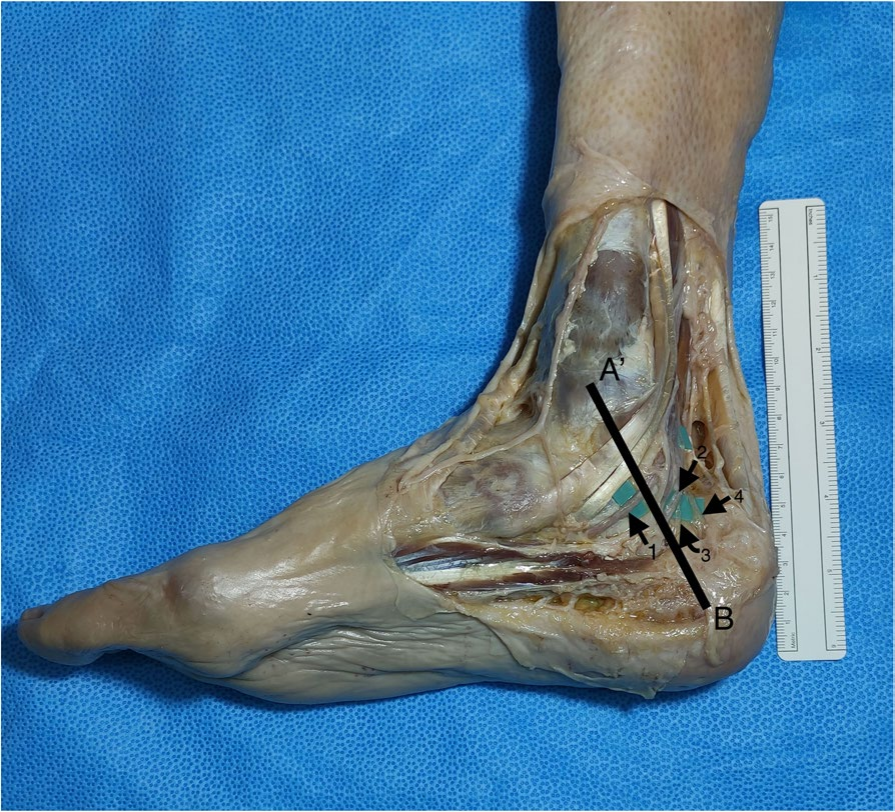
Medial view of the right distal part of the leg, ankle and foot. The Dellon–McKinnon malleolar-calcaneal line (DML) length was measured and the relationship, relative position and distance, with the tibial nerve (TN) branches was determined. A’: center of the medial malleolus (MM); B: tip of the medial process of the calcaneal tuberosity at its greatest distance from the MM; 1: Medial plantar nerve (MPN); 2: Lateral plantar nerve (LPN); 3: Baxter’s nerve (BN); 4: Medial calcaneal nerve (MCN)

As routine in our Unit, appropriate dissection techniques were performed by using proper dissection tools in order to achieve the objectives of the study [12–14]. The specimens were placed in a supine position and the medial side of the distal part of the leg, ankle and foot were carefully dissected in order not to disturb the normal anatomy of the medial region of the ankle [15]. A posteromedial approach to the ankle was used (Fig. 2). The skin incision started approximately 8 cm proximal to the medial malleolus (MM) and extended nearly to the level of the talocalcaneonavicular joint (Fig. 2a). Small incisions approximately perpendicular to it were done to facilitate the retraction of the skin. The skin was carefully retracted leaving the superficial fascia intact. Then, the superficial fascia was carefully removed in order to preserve the vascular and nervous structures lying in it (Fig. 2b). The deep fascia, and a band-shaped thickening of it, the FR, were identified (Fig. 2c). The deep fascia was removed leaving the FR intact, and the proximal part of the AH was identified and dissected (Fig. 2c). The FR was detached from its tibial fixation in order to expose the structures that enter the sole deep to it, that are, from medial to lateral, the tendons of tibialis posterior and flexor digitorum longus, the posterior tibial vessels, the TN and the tendon of flexor hallucis longus (Fig. 3) [12, 13]. Then, the neurovascular bundle was identified. The vascular structures were isolated and, whenever necessary, carefully pushed apart, without cutting them off, in order to better identify and dissect the nervous structures (TN, LPN, MPN, BN, MCN) without moving it from its proper position (Fig. 4).

The measurements presented were based on the Heimkes Triangle (HT) and the Dellon–McKinnon malleolar-calcaneal line (DML) (Fig. 3). The triangle was first described in the work of Heimkes et al. and counted with the following vertices: the tip of MM (point A), the tip of the medial process of the calcaneal tuberosity at its greatest distance from the MM (point B), and the tuberosity of the navicular (point C) [7]. The DML was initially described in the work of Dellon and Mackinnon and is drawn from the center of the MM (point A’) to the tip of the medial process of the calcaneal tuberosity at its greatest distance from the MM (point B) [16].

Every landmark described can be felt by direct palpation and visualized without the use of microsurgical instruments and optical magnification. All the measurements were performed with the ankle in anatomic position stabilized by an assistant and using a flexible surgical ruler (Fig. 4).

DML length was measured, the center was marked and the relationship (relative position and distance) with the TN division and BN origin was determined (Fig. 4). The distance of the MPN, LPN, BN and MCN from the center of the MM (point A’), on top of the DML was measured (Fig. 4). The distance from the tip of the medial process of the calcaneal tuberosity at its greatest distance from the MM to the MPN, LPN and BN, on top of the BC line (distal edge of the HT used to simulate the point where neurologic structures cross the medial septum/AH) was also measured (Fig. 3).

**Fig. 5:**
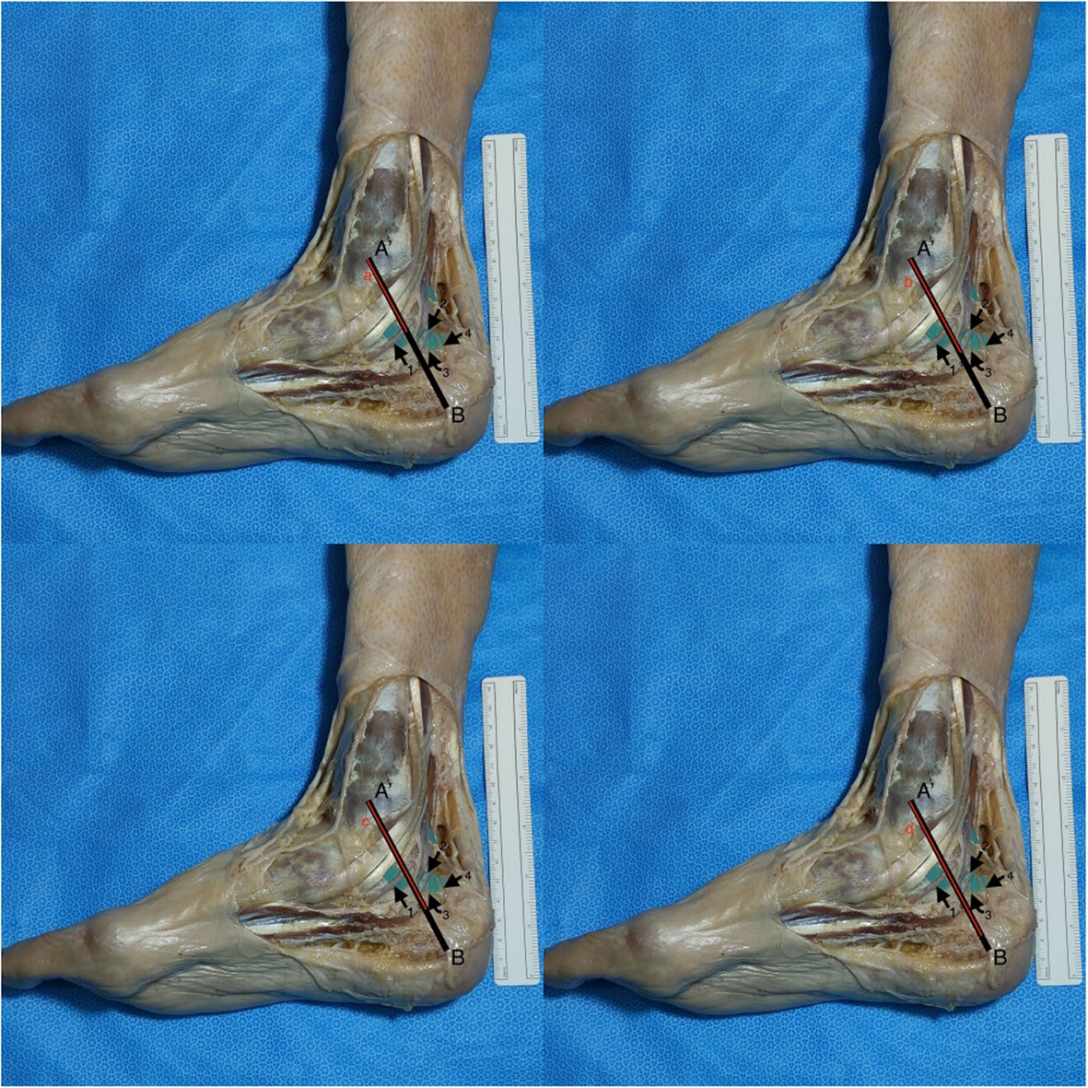
Pearson’s correlation coefficient was used to determine the correlation between: DML length (A’ to B) and the distance between the center of the MM and the point where the MPN crosses the DML, line a, the point where the LPN crosses the DML, line b, the point where the BN crosses the DML, line c, and the point where the MCN crosses the DML, line d (not evident on the displayed image so the line shown is merely a representation)

The statistical analysis was performed using *SPSS Statistics 26* (Armonk, NY: IBM Corp.). Mean and standard deviation values were calculated for all the measurements. Pearson’s correlation coefficient was used to determine the correlation between 1) DML length and 2) the distance between the center of the MM and the point where the DML is crossed by either the a) MPN, b) LPN, c) BN, or d) MCN (Fig. 5). The significance level was set at ɑ = 0.01.

## Results

The DML line had a mean length of 74 mm (range 50-98 mm). We verified that there was a tendency for taller specimens to have longer DMLs. However, the Pearson’s coefficient (ρ) between “height” and “DML length” was 0.317; *p* = 0.131.

In 21out of 24 (87,5%) feet, the origin of MPN and LPN was proximal to DML and on the other 3 (12,5%), it appeared on top of the DML. The distance between the TN division and the center of the DML was, on average, 18 mm with a maximum of 40 mm. There was no correlation between this distance and the length of DML or the height of the body (Table 1).

**Table 1:** Correlation between “Distance between the TN division/ BN emergence and the center of the DML” and “Length of DML” and “Height of the subject”

		Length of DML	Height of the subject
Distance between the TN division and the center of the DML	**Pearson Correlation**	0.057	- 0.163
	**Significance**	0.792	0.447
Distance between the BN emergence and the center of the DML	**Pearson Correlation**	- 0.282	- 0.224
	**Significance**	0.182	0.293

By applying the same measurements for the BN, we found 12 (50%), out of 24, emerging proximal to the DML. Nine (37,5%) were dividing distal to DML and the remaining 3 (12,5%) were overlying the DML. The average distance between the BN emergence and the DML’s midpoint was 15 mm (range 5-25 mm) for the nerves dividing proximal to DML and 9 mm (range 2-15 mm) for the ones emerging distal to DML (Fig. 4). There was no significant correlation between the distance from the midpoint of DML and the emergence of BN and DML’s length or body’s height (Table 1).

On the DML we measured the distance of the MPN, the LPN, the BN and the MCN from the center of the MM. The average distance was 33 mm (range 20-50 mm) for the MPN; 39 mm (range 20–57 mm) for the LPN; 43 mm (range 30-58 mm) for the BN and 53 mm (range 39–72 mm) for the MCN.

Using Pearson’s correlation coefficient a strong correlation was documented, not only between the DML length and the distance between the center of the MM (Fig. 5) and the point where the LPN crosses the DML (ρ = 0.910, *p* <  0.001), but also, between DML length and the distance between the center of the MM and the point where the MPN crosses the DML (ρ = 0.866, p <  0.001) (Table 2). Additionally, a very strong correlation was also noted between the DML length and the distance between the center of the MM and the point where the BN crosses the DML (ρ = 0.970, p <  0.001). The same coefficient was used to analyze the correlation between the DML length and the distance between the center of the MM and the point where the MCN crosses the DML (ρ = 0.762, *p* <  0.001) (Table 2).

**Table 2:** Correlation between the length of the DML (distance from A’ to B) and the distance from the center of the medial malleoli (point A’) to the MPN, LPN, BN and MCN on top of DML

		Distance of MPN from A’ on top of DML	Distance of LPN from A’ on top of DML	Distance of BN from A’ on top of DML	Distance of MCN from A’ on top of DML
Distance from A’ to B	**Pearson Correlation**	0.866	0.910	0.970	0.762
	**Significance**	< 0.001	< 0.001	< 0.001	< 0.001

The distance from the medial process of the calcaneal tuberosity, on top of the BC line, (Fig. 4) was, on average, 31 mm (range 22-45 mm) for LPN, 44 mm (range 30-70 mm) for MPN and 24 mm (range 15-35 mm) for BN.

If we defined a central point, 30 mm from the medial process of the calcaneal tuberosity on top of the AH, for the LPN, we would be able to find the nerve within a 5 mm radius in 19 out of 24 feet. The same radius for MPN and BN with central points of 45 mm and 25 mm, respectively, would find the nerve in 13 out of 24 feet for the first one and 17 out of 24 feet for the second. However, if a 7 mm radius for MPN is used, it would include the nerve in 17 out of 24 feet.

## Discussion

We dissected 24 Thiel embalmed feet in order to better understand the anatomical topography of the ankle’s medial side. The origin and distribution of TN, MPN, LPN, MCN and BN were registered according to specific landmarks and possible compression points.

Chronic heel pain syndrome is a commonly encountered condition among surgeons dedicated to foot and ankle [1, 2, 17, 18] but an accurate diagnosis is not always easy. A complex regional anatomy opens the door to many etiologies like space occupying lesions, bony prominences compression, posttraumatic or perineural fibrosis, muscle anomalies, among others that, altogether, make the neurological compression one of the most cited causes. However, in a considerable percentage of cases, the true cause can remain unknown [2, 19, 20].

The TT is a fibro-osseous tunnel beneath the FR, behind and inferior to the MM [7, 21]. Rosson et al. and Tekin et al. stated that, under FR, there were increased pressures leading to the clinical findings on TTS, only relieved with the retinaculum release [21, 22]. Also, some foot and ankle positions like increased eversion, dorsiflexion, or combined dorsiflexion-eversion may significantly increase TN tension on the TT [23–25]. Therefore, the compression of the TN and/or its main branches on the medial side of the ankle under the FR has been already established as a cause of the symptoms on TTS.

In 1984, Dellon and Mackinnon showed that in 90% of their cases, the bifurcation of the TN was within 1.0 cm of the DML and that in 95% of the cases it was under the FR [16]. More recently, Mattos et al. [26] and Gamie et al. [27] stated, on their work, that the MPN has its origin proximal to the DML in 95 and 100% of the cases, respectively. Moreover, Moroni et al. [8] presented an average distance of 16,4 mm from the center of DML to the TN bifurcation, a result close to the 18 mm that we obtained. However, despite the average distances being close to each other, the values found within each study are very variable ranging from 40 mm to -10 mm on Moroni’s [8] and from 0 mm to 40 mm on ours.

The FR starts, approximately, 20 mm proximal to DML and ends 20 mm distal to it [16]. We found the division of the TN within less than 20 mm from the center of the DML in 14 (58%) of our specimens and Moroni et al. on approximately 73% [8]. Bilge et al. proposed a classification for the TN division according to a reference line (1 cm width) from the tip of the MM to the medial process of the calcaneal tuberosity [28]. Type I represented a bifurcation proximal to the line, Type II overlying it and Type III distal to it. Their work concluded that 84% of the cases were type I, 12% type II and 4% type III [28]. This data is in close relation with ours, where, according to the mentioned classification, 88% of the cases were type I and 12% type II.

Being a site of compression and a well-defined target in TTS’s surgical treatment, the TT demands a good understanding of the anatomy behind the syndrome. Our results corroborated previous works that suggested a division of the TN at a variable distance from the superior limit of the FR [8, 16, 26, 27]. It is more common to find this division proximal to the FR which, in turn, suggests that it will be more common to find a compression of the main branches under the FR than the TN itself. Further in vivo studies are needed to understand if there is any relation between the occurrence of a TTS and the division of the TN outside the FR.

We tried to identify the relationship of the neural structures with anatomical landmarks that a surgeon can use in a daily basis and found that, on average, we could encounter the MPN, the LPN, the BN and the MCN, at 33 mm; 39 mm; 43 mm and 53 mm, respectively, from the center of the MM, on top of the DML. Moroni et al. did similar measurements and found the MPN, the LPN and the BN, on average, at 37 mm, 44 mm, 48 mm, respectively [8]. The values obtained by us and Moroni et al. are similar but we still can find a great variability among the reported distribution of TN and its branches on the current literature [7, 16, 29].

We found that there was a strong correlation between the DML’s length and the point where we encountered the MPN, the LPN the BN and the MCN. This means that the distance from the MM and the point where we find the MPN, the LPN and the BN under the FR is strongly correlated with the total distance from the MM and the medial process of the calcaneal tuberosity and, therefore, the size of the ankle. On that account, we hypothesize that the pathway for these nerves could be more predictable than we previously thought if we consider the size of the ankle. Heimkes et al. described that, at the inferior retinaculum limit, both MPN and LPN enter two separate fibro-osseous tubes [7]. We put on consideration that these tubes may condition a more regular distribution of the branches proximally. In this domain, Mattos et al. also found that MPN, along with MCN, was the nerve with the most constant distribution in relation to the DML [26]. There is still limited information on the literature about predictability of the BN trajectory however, our work can give some new insights on this topic, suggesting that distance from the MM where we find BN in the ankle is also strongly correlated with the size of the ankle itself.

This notion of a strong correlation can be extremely important as most works, ours included, report average distances where we can find the nerves on the medial side of the ankle. However, we should be aware that this distance is strongly correlated with the total distance from the MM to the medial process of the calcaneal tuberosity and, therefore, when we analyze these values we should always take into consideration the relation with total length of the DML and not the absolute value. We also believe that this information can shed some light into the way we analyze the trajectory of these nerves.

Singh and Kumar stated that the medial septum, a dorsal extension of the medial border of the central plantar aponeurosis, also referred to as the deep fascia of the AH in some studies, could be the most important compressive structure, besides the FR, behind TTS [5]. Ghosh et al. also stated that both the LPN and MPN could be entrapped under the AH and this possibility should be excluded if no compression at the FR was found [30]. Therefore, we tried to address its importance by checking the distribution of the MPN, the LPN and the BN along the distal edge of the Heimkes Triangle, simulating the AH and the septum. We found them at 44 mm, 31 mm and 24 mm, respectively, from the medial process of the calcaneal tuberosity. Singh and Kumar also reported mean distances of points of penetration of TN branches through the foot septae from the posterior surface of the calcaneus of 56 mm for the MPN, 41 mm for the LPN and 29 mm for the BN [5]. The results obtained by us and Singh and Kumar are quite different. However, few works on the literature addressed these measurements and the data available is still scarce to determine if these structures have, in fact, a variable and unpredictable trajectory or if, on the other hand, could have a foreseeable passage. Moreover, slightly different anatomic landmarks were used between our (same as Dellon and Mackinnon [16] used) and Singh and Kumar’s [5] works which may also contribute to the differences found.

Surgical treatment of TTS is reserved for patients who do not respond to conservative measures. Unpredictable results may characterize surgical options, with some authors reporting relief of symptoms in only 50% of cases, others with poor results at long-term follow-up in more than 50% [2, 6]. Many studies focus on the necessity of including the nerve’s crossing through AH and medial septum on the surgical strategy for treating the TTS, but the best surgical strategy is highly controversial. Mullick and Dellon reported excellent results in 82% of the patients, after a 3 years follow-up, with decompression of four ankle tunnels, the FR and the tunnels for the MPN, the LPN and the MCN [6]. Thus, the distal tarsal tunnel seems to be undeniably connected to TTS despite remaining a challenging anatomical region. Our work tries to make it easier to understand the relationship between some landmarks and the nerves that need to be released under the AH and medial septum.

More recently, an ultrasound-based approach to the TTS was introduced with similar outcomes compared to open releases, minimizing soft tissue dissection, potential wound complications like infections and scars, with reducing recovery time and avoiding offloading [19, 31, 32]. The procedure is based on accurate identification of neural structures and a minimally-invasive cut of the FR and AH fascia [19, 31]. The techniques described are quite demanding and the surgeon must have a clear understanding of how the nerves are displayed, especially when releasing the distal TT [31].

Endoscopic procedures are also coming to light and precise anatomical references are needed so that either the correct placement of the portals and the safety of the technique in relation to the neurologic structures are assured [33]. The two portals endoscopic procedure was first described by Day and Naples [34]. On their study, the proximal portal was located over the palpable FR, about 1 cm proximal to the MM [34]. El Shazly et al. stated that the main indication for the endoscopic technique would be idiopathic proximal TTS with main trunk compression and proposed a revised proximal portal, 4 cm from the MM, that would facilitate the retinaculum release [33]. Our results could be in line with this new perspective about the proximal portal, suggesting that surgeons must take into consideration that the division of the TN may be variable and that can be found as proximal as 40 mm from the DML. This knowledge would be fundamental for safe portal placement and for a correct visualization of this division.

Awareness of possible points of division and trajectories is of tremendous importance for procedures like nerve blocks of the tibial nerve or its branches. Tibial nerve block was reported as a safe and effective method for controlling pain after outpatient surgery of hallux valgus [35]. The average distances from specific anatomical landmarks for the TN and its branches that are shown in our work gives important information for safe and effective blocks.

Lee at al. stated that an important percentage of nerve injuries after ankle replacement were due to excessive stretching during retraction, inadequate nerve release or improper protection during the incision [36]. Once again, an adequate knowledge of the position of the tibial nerve and its main branches according to specific landmarks like MM or the medial process of the calcaneal tuberosity will provide a greater security approaching this area.

Our study had some limitations. As it was a work based on cadaveric specimens the possible sample size was small (24 feet) and therefore our conclusions are limited. Moreover, all the subjects included were Caucasians and with more than 60 years old which can condition, in some degree, the results obtained. Additionally, we are aware that our study has also other limitations that are generally observed in studies that are performed in cadavers. However, we had done everything possible to minimize, at least in part, these limitations. One limitation is related to the changes induced by death and fixation procedures in the volume and trophicity of tissues. We tried to minimize this problem by using bony landmarks. Furthermore, we have measured some reference parameters and the results were very similar to the ones reported earlier in the scientific literature, a point that unequivocally provides robustness to our results. On the other hand, we highlight that our study was optimized by using full-body cadavers. In fact, in this way, all the studied structures, particularly the nerves, were intact. Additionally, we re-emphasize that all dissection procedures were carefully performed to maintain, as much as possible, the normal relationships between structures. Finally, another limitation of the present study is the absence of clinical information related to the studied specimens which prevented any correlation between anatomical and clinical factors.

## Conclusion

With this work we were able to show a topographical relation between neural structures and possible compression points which will allow a better anatomical interpretation of the medial heel region when an invasive procedure is needed. However, there is still a lot of work to do in terms of understanding how the anatomy can influence TTS. We were able to suggest that, despite all the variability concerning the neural anatomy around the medial heel region, some structures can be found with a considerable regularity in a specific location, a regularity that can be very handy when, for example, a surgical treatment or a nerve block procedure is required. With new techniques allowing better results, more anatomical studies are required to indulge safer procedures.

## Data Availability

The datasets used and/or analyzed during the current study are available from the corresponding author on reasonable request.

## Abbreviations

AH: Abductor Hallucis
BN: Baxter’s Nerve
DML-Dellon: McKinnon malleolar-calcaneal line
FR: Flexor Retinaculum
HT: Heimkes Triangle
LPN: Lateral Plantar Nerve
MCN: Medial Calcaneal Nerve
MM: Medial Malleolus
MPN: Medial Plantar Nerve
ρ: Pearson’s Correlation Coefficient
TN: Tibial Nerve
TT: Tarsal Tunnel
TTS: Tarsal Tunnel Syndrome
